# The effect of pretreatment with hydroalcoholic extract of *Alpinia officinarum* rhizome on seizure severity and memory impairment in pentylenetetrazol-induced kindling model of seizure in rat

**DOI:** 10.3934/Neuroscience.2019.3.128

**Published:** 2019-08-16

**Authors:** Kamal Solati, Zahra Rabiei, Samira Asgharzade, Hossein Amini-Khoei, Ali Hassanpour, Zahra Abbasiyan, Maryam Anjomshoa, Mahmoud Rafieian-Kopaei

**Affiliations:** 1 Medical Plants Research Center, Basic Health Sciences Institute, Shahrekord University of Medical Sciences, Shahrekord, Iran; 2 Cellular and Molecular Research Center, Basic Health Sciences Institute, Shahrekord University of Medical Sciences, Shahrekord, Iran

**Keywords:** *Alpinia officinarum*, seizure, pentylentetrazol, memory

## Abstract

The aim of present study is to investigate pretreatment with hydroalcoholic extract of *Alpinia officinarum* rhizome on the severity of epilepsy and memory impairment in rat. In this experimental study, rats were randomly assigned to seven groups. Control group and negative control group were intraperitoneally injected with normal saline and PTZ, respectively, for 10 days. The intervention groups received *A*. *officinarum* extract at different doses (50, 100 and 150 mg/kg) 30 minutes before PTZ injection. *A*. *officinarum* extract treatment in rats with PTZ-induced kindling exerted significant increase in seizure latency and significant decrease in the frequency of total body seizure, frequent spinning, and jumping. Flumazenil significantly inhibited the antiepileptic effects of A. *officinarum* extract in the rat receiving the extract at 150 mg/kg. *A*. *officinarum* extract can inhibit PTZ-induced seizure and memory impairment, and therefore can be considered as a potent agent which warranted further research to clarify its effects.

## 1. Introduction

Epilepsy refers to a spectrum of chronic neurological disorders characterized by epileptic seizures. These attacks may be very mild and undiagnosable or long-term with severe trembling. About 1% of the world population (65 million) suffers from epilepsy, approximately 80% of whom live in developing countries. The hippocampus is one of the major regions of the brain involved in epilepsy and plays an important role in the learning process and memory, especially in acquiring spatial memory. Lesions in the region cause memory loss and spatial memory impairment [1]. The frequent incidence of epileptic seizures significantly declines learning rate and memory in patients with epilepsy. It has been demonstrated that pentylenetetrazol (PTZ)-induced chemical kindling can lead to learning disorders in laboratory animals. The hippocampus is not only effective on memory and learning, but also on the onset, generalization, and termination of seizure. The brain neurodegeneration, especially in the CA1 region of the hippocampus, and change in the function of changeable synapses that store the information, are a possible explanation of the observed learning disorder following kindling [2]. PTZ is a chloride channel blocker of choice that binds to the GABA_A_ receptor complex, and is a chemical inducer of seizure used to study antiepileptic drugs (AEDs) in animal models. It has been argued that PTZ exerts adverse effects on neuronal membrane, affects potassium and calcium channels, releases intracellular calcium ion reserves, and reduces the neurotransmitter-induced chloride conductance [3]. Oxidative stress is likely to play a role in the onset and progression of epileptic seizures. It has been reported that epileptic seizures are associated with the hemostatic imbalance of antioxidants and oxidants [4]. Animal studies have indicated that PTZ-induced epileptic seizures are associated with significant increase in lipid peroxidation and significant decrease in the antioxidant activity of superoxide dismutase (SOD) and catalase (CAT) as well as glutathione levels in the red blood cells, liver, and brain [4],[5]. The use of antioxidant compounds to cope with epileptic seizures has recently attracted attention and the beneficial effects of certain antioxidants have been demonstrated. Galangal, botanically referred to as *Alpinia officinarum*, is from the family Zingiberaceae and native to Southeast Asia and China. In Iranian traditional medicine, *A*. *officinarum* is used to treat common cold, to improve potency and memory, digestive disorders and stomachache, low back pain, rheumatism, and sciatica. *A*. *officinarum* is also used as an emetic, anti-flatulence, and anti-muscle spasm agent. To date, the therapeutic effects of *A*. *officinarum* have been investigated, and the antioxidant [6], anti-inflammatory [7], analgesic [8], hypolipidemic [9], and antimicrobial [10] effects of this plant have been confirmed. Various compounds have been identified in *A*. *officinarum* rhizome by chromatographic techniques, including 1, 8-cineol, methyl cinnamate, galangin, 3-O-methyl galangin, kaempferide, alpinin, galangol, and diarylheptanoids. Galangin and 3-o-methyl galangin are two main compounds of *A*. *officinarum* rhizome. Galangin is a flavonoid that is found in abundance in *A*. *officinarum* rhizome. Galangin has different biological activities, including anti-mutagenic, anti-oxidant, and metabolic enzyme-modulating activities. Besides that, 3-o-methyl galangin has been reported to exhibit hypolipidemic effects [11]. There are several methods to investigate the seizure, antiseizure agents and related disorder to seizure in the rodents including kindeling, tonic, clonic seizure and etc. In this study we aimed to evaluate the protective effect of hydroalcoholic extract of *Alpinia officinarum* rhizome on memory function as well as seizure sensevity according to the previous study PTZ-induced model of seizure is an appropriate paradigm to evaluate these behaviors.

## 2. Material and method

### 2.1. The preparation of A. officinarum extract

*A*. *officinarum* was purchased from a reliable grocery and botanically and systematically identified as *A*. *officinarum*; then, 1000 g of its dried root was dissolved in 2000 ml of 70% ethanol. After the solution was left at room temperature for 48 hours, it was filtered and its solvent was isolated in a rotary evaporator. Then, the resulting extract was completely dried at 40 °C before preparing the required concentrations.

Chemicals: PTZ, diazepam and flumazenil were purchased from Sigma-Aldrich (St. Louis, MO, USA). The chemical formula of diazepam and flumazenil is given below.

**1.**
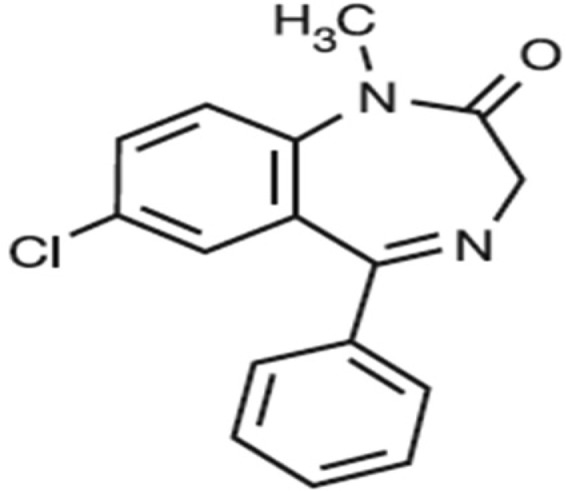
Diazepam

**2.**
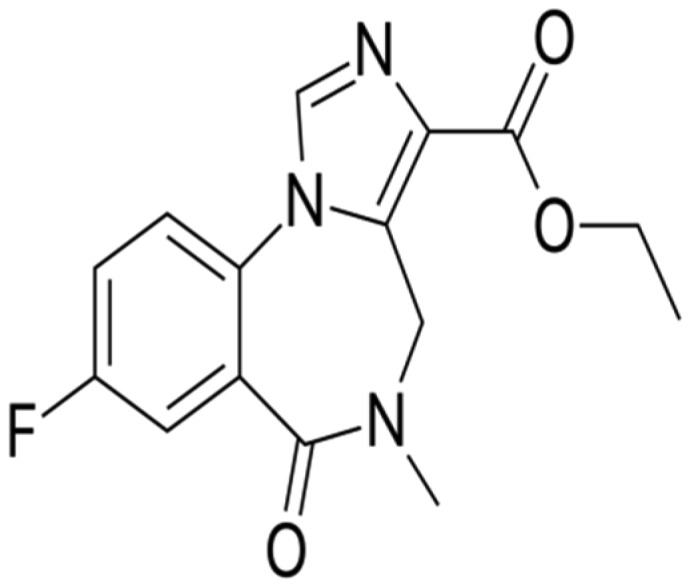
Flumazenil

Male Wistar rats weighing 200 ± 30 g were used in this study. The rats were caged in groups of 10 each in the Animal House of the Shahrekord University of Medical Sciences less than 24 °C temperature and 12-h dark/12-h light cycle with free access to water and food. All stages of experimentation were carried out in accordance with the regulations of the University and the Guide for the Care and Use of Laboratory Animals of National Institutes of Health and Guide for the Care and Use of Laboratory Animals (8th edition, National Academies Press). Full efforts were made to diminish the use of animals and to improve their wellbeing.

The rats were randomly assigned to seven groups of eight each as follows:

Control group (G1) and negative control group (G2) intraperitoneally received, respectively, normal saline and PTZ every 48 hours for 10 days;

Groups 3 (G3) and 4 (G4) and treatment group (G5) intraperitoneally received PTZ every 48 hours for 10 days, and 30 minutes later, *A. officinarum* extract at 50, 100 and 200 mg/kg daily;

Positive control group (G6) intraperitoneally received PTZ for 10 days, and 2 mg/kg diazepam on 10th day 30 minutes before PTZ treatment;

Group 7 (G7) intraperitoneally received *A. officinarum* extract 30 minutes after PTZ treatment for 10 days and 2 mg/kg flumazenil 10 minutes before intraperitoneal treatment with 150 mg/kg *A*. *officinarum* on the 10th day.

To induce epilepsy, 35 mg/kg of PTZ was intraperitoneally injected daily every 48 hours every other day for 9 days.

The behavioral tests were conducted during the second 5 days. On the 10th day, 60 mg/kg of PTZ was administered. Injections were conducted for 10 days and the severity and rate of seizure were recorded during the 30 minutes after the injection. Diazepam and flumazenil were administrated in single dose on the last day of the treatment. In general, after intraperitoneal injection of PTZ, the stages of epilepsy consist of:

Stage 1: Head ticks;

Stage 2: The seizure of the entire body and rearing;

Stage 3: Tonic seizures;

Step 4: Frequent spinning and jumping. The animal either dies or restores normal conditions after stage 4. All four steps do not necessarily occur and some steps may occur so quickly that they are not detected.

### 2.2. Tail suspension test (TST)

To conduct TST, two 70-cm metal anchors are used. The anchors are longitudinally stretched between two metal bases by a 50-cm rope. The animal's tail is closed by a rope and the animal is hanged up from its tail. First, the rat begins to jerk and then completely becomes inactive and motionless. The duration of motionlessness indicates immobility time.

### 2.3. Testing passive avoidance memory using shuttle box

The shuttle box was used to test the passive avoidance memory. This apparatus includes a bright chamber connected to a dark chamber by a guillotine door. Electric shocks are exerted by a separate stimulator to the grid floor of the shuttle box. This test was performed on each rat within four days. To adapt to the apparatus, each rat was allowed 5 min to explore it on the first and second days. An acquisition test was conducted on the third day. The rats were separately left in the bright chamber, and after a 2-minute adaptation, the door was opened, the rats entered the dark chamber, the door was closed, and an electrical shock (1 mA/second) was exerted to the rats so that they only paddled. In this test, initial latency was considered latency to enter the dark chamber and its duration was recorded. Twenty four hours later, each rat was left in the bright chamber, and the interval between being left in the bright chamber and entering the dark chamber was measured (up to 60 seconds) and considered the second latency.

### 2.4. Measuring the antioxidant capacity of the serum and the brain

Three solutions were used to measure serum antioxidant capacity consisting of buffer (1.55 ml of sodium acetate and 8 ml of concentrated acetic acid reaching 500 ml volume using distilled water); iron chloride solution (270 mg of iron (III) chloride reaching 50 ml volume using distilled water; and triazine (47 mg of triazine dissolved in 40 ml of 40 mM hydrochloric acid).Stock solution was prepared by adding 10 ml of solution 1 to 1 ml of solution 2 and 1 ml of solution 3. Twenty five µl of serum sample or brain homogenate was added to 1.5 ml of the stock solution and the optical absorbance was read at 593 nm after the stock solution was left at 37 °C for 10 minutes.

### 2.5. Measurement of lipid peroxide levels in serum and brain tissues

Two hundred µl of tissue homogenate/serum was mixed with 1.5 ml of 20% acetic acid, 1.5 ml of 0.8% TBA and 200 µl of 8.1% SDS. The mixture was made up to 4 mL with distilled water and heated in a boiling water bath for 60 minutes. After cooling under tap water, 1 ml of distilled water and 5 ml of n-butanol/pyridine were added to the reaction mixtures and shaken vigorously. Then, the resulting solutions were centrifuged at 4000 rpm for 10 min and the optical absorbance of the supernatant at 532 nm was recorded. Lipid peroxide levels were determined using standard calibration curve and expressed as a micromol of malondialdehyde.

### 2.6. Statistical analysis

All data analysis was conducted by SPSS 16. First, Kolmogorov-Smirnov test was used to investigate the normality of the data distribution and Levene's test to investigate the homogeneity of variances. One-way analysis of variance (ANOVA) and Tukey's test were used to investigate the significance of difference between the treatments and mean values, respectively. Data were expressed as mean ± standard error, and *P* < 0.05 was considered significance level.

## 3. Results

### 3.1. Rat's survival rate

**Figure 1.**
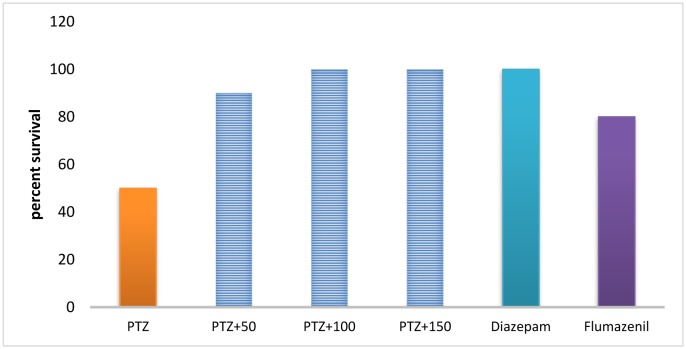
The survival percentage in different groups. # significant difference compared to pentylenetetrazol-receiving group (*P* < 0.05).

The survival rates in the PTZ, diazepam, PTZ + the extract (50, 100 and 150 mg/kg), and flumazenil groups were 50%, 100%, 90%, 100%, 100%, and 80%, respectively. Fisher's exact test showed a significant difference in survival rate between the groups (*P* < 0.001) (Figure 1).

### 3.2. First seizure latency

**Figure 2.**
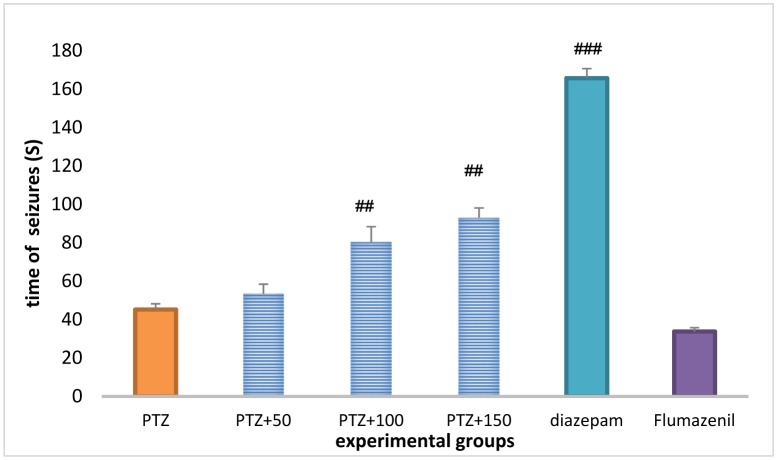
First seizure latency in different groups. ^###^Significant difference when compared to pentylenetetrazole receiving group (*P* <0.001); ^##^ Significant difference compared to pentylenetetrazol-receiving group (*P* < 0.01).

Treatment with *A*. *officinarum* extract (100 and 150 mg/kg) caused a significant increase in the first seizure latency in the PTZ-receiving rats (*P* < 0.001). The first seizure latency was significantly higher in the diazepam group than in the PTZ group (*P* < 0.001), and significantly lower in the flumazenil + extract (150 mg/kg) group than in the group receiving the extract (150 mg/kg) (*P* <0.001), but no significant difference was observed between the flumazenil + extract (150 mg/kg) group and the PTZ group (Figure 2).

### 3.3. The frequency of head ticks

**Figure 3.**
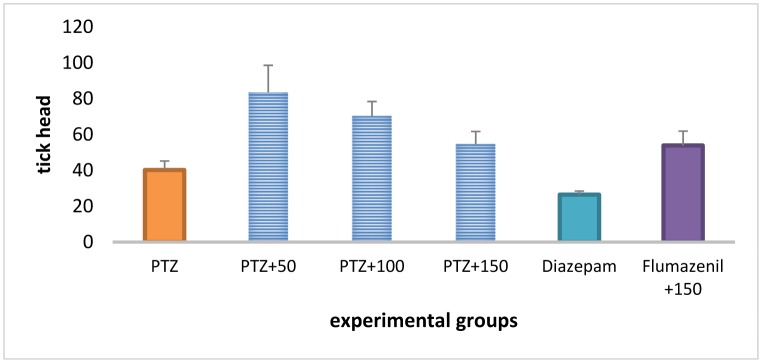
The total frequency of head ticks in different groups.

Treatment with *A*. *officinarum* extract (100 and 150 mg/kg) and diazepam produced no significant effect on the total frequency of head ticks in the PTZ-receiving rats. (Figure 3).

### 3.4. The total frequency of the entire body seizures

**Figure 4.**
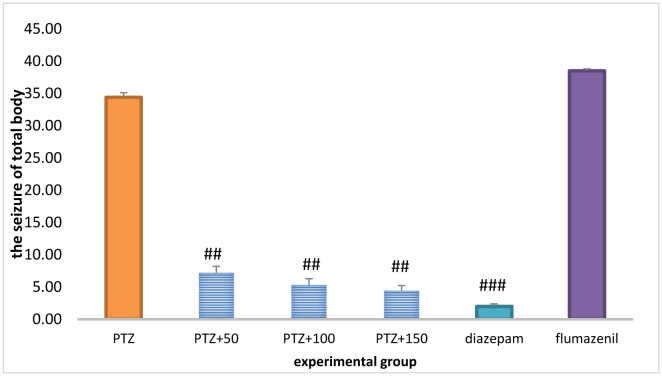
The total frequency of the entire body seizures in different groups. ## Significant difference compared to pentylenetetrazol-receiving group (*P* < 0.01); ### Significant difference compared to pentylenetetrazol-receiving group (*P* < 0.001) (150 mg/kg) (*P* < 0.001).

Treatment with diazepam + the extract (50, 100 and 150 mg/kg) in the PTZ-receiving rats significantly reduced the total frequency of the entire body seizures (*P* < 0.001). The total frequency of the entire body seizures was significantly higher in the flumazenil + extract (150 mg/kg) group than in the extract (150 mg/kg) group (*P* < 0.001), but was not significantly different compared to the PTZ-receiving group (Figure 4).

### 3.5. The total frequency of tonic seizures

**Figure 5.**
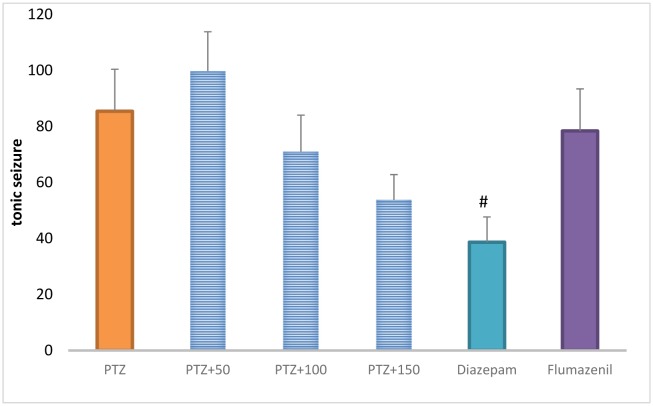
The total frequency of tonic seizures in different groups. #Significant difference compared to pentylenetetrazol-receiving group (*P* < 0.05).

The total frequency of tonic seizures was not significantly different between the extract (100 and 150 mg/kg)-receiving groups and the PTZ-receiving group. The frequency of tonic seizures was significantly lower in the diazepam-receiving group than in the PTZ-receiving group (*P* < 0.05) (Figure 5).

### 3.6. The total frequency of repeated spinning and jumping

**Figure 6.**
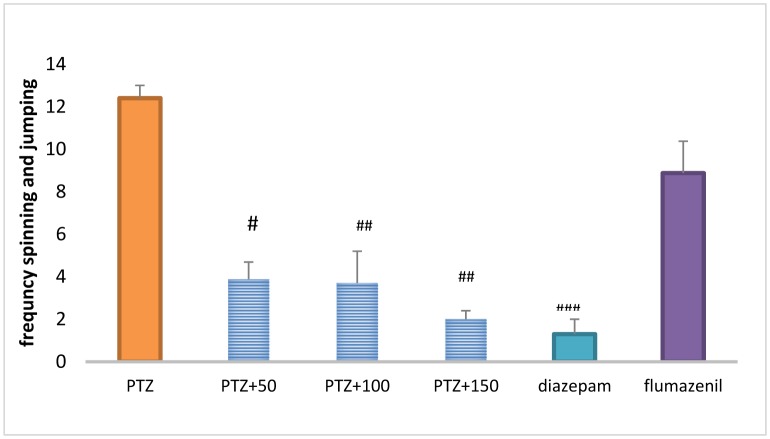
The total frequency of repeated spinning and jumping in different groups. ### Significant difference compared to pentylenetetrazol-receiving group (*P* < 0.001); ## Significant difference compared to pentylenetetrazol-receiving group (*P* < 0.01); #Significant difference compared to pentylenetetrazol-receiving group (*P* < 0.05).

Treatment with diazepam + extract (50, 100 and 150 mg/kg) caused a significant decrease in the total frequency of repeated spinning and jumping in the PTZ-receiving rats (*P* <0.01). Repeated spinning and jumping was significantly higher in the flumazenil + extract (150 mg/kg) group than in the extract group (150 mg/kg) (*P* < 0.01) (Figure 6).

### 3.7. Immobility time in TST

**Figure 7.**
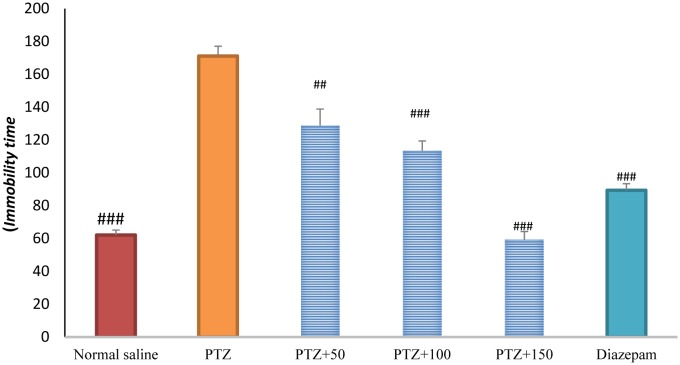
Immobility time in tail suspension test in different groups. ### Significant difference compared to pentylenetetrazol-receiving group (*P* < 0.001); ## Significant difference compared to pentylenetetrazol-receiving group (*P* < 0.01).

Immobility time was significantly lower in the control group than in the PTZ-receiving group (*p* <0.001). Treatment with diazepam + extract (50, 100, and 150 mg/kg) caused a significant decrease in immobility time in the PTZ-receiving rats (*P* < 0.01) (Figure 7).

### 3.8. Initial latency in shuttle box test

**Figure 8.**
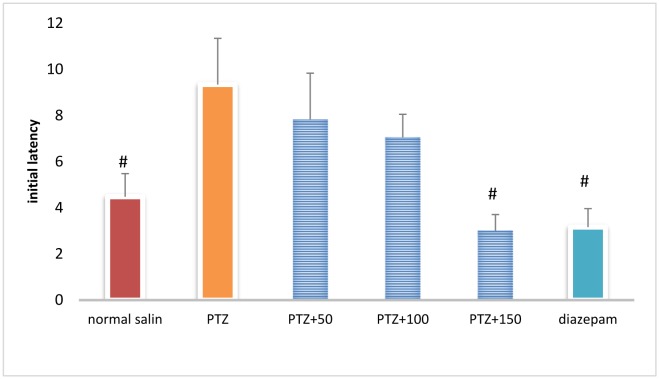
Initial latency in the shuttle box test in different groups. #Significant difference compared to pentylenetetrazol-receiving group (*P* < 0.05).

Initial latency was significantly different between the control group and PTZ-receiving group (*P* < 0.05). Initial latency was lower in the PTZ + extract (150 mg/kg)-treated group and diazepam group than in the PTZ-treated group (*P* < 0.05) (Figure 8).

### 3.9. Secondary latency in shuttle box test

**Figure 9.**
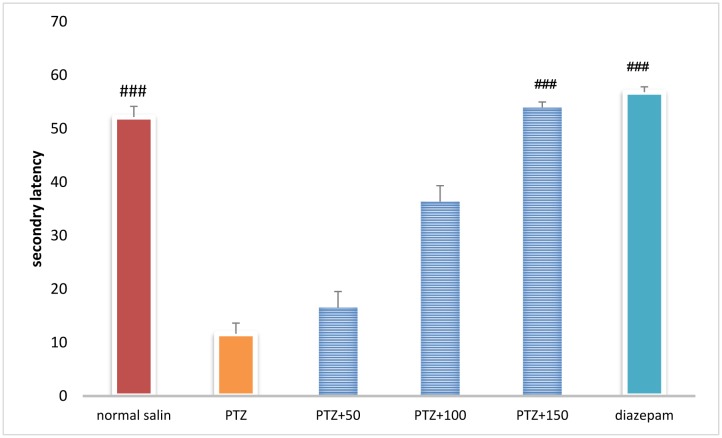
Secondary latency in shuttle box test in different groups. ### Significant difference compared to pentylenetetrazol-receiving group (*P* < 0.001).

Secondary latency was significantly higher in the control group than in the PTZ-receiving group (*P* < 0.001). Treatment with diazepam + extract (100 and 150 mg/kg) caused a significant increase in secondary latency in the PTZ-receiving rats (*P* < 0.001) (Figure 9).

### 3.10. Serum antioxidant capacity

**Figure 10.**
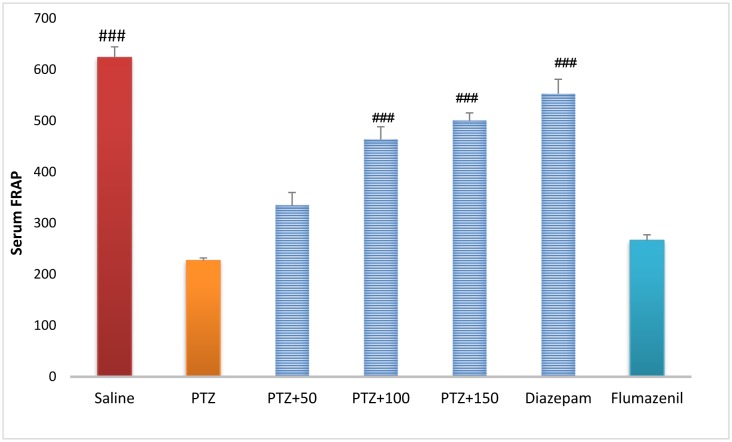
Serum antioxidant capacity in different groups. ### Significant difference compared to pentylenetetrazol-receiving group (*P* < 0.001); ## Significant difference compared to pentylenetetrazol-receiving group (*P* < 0.01).

Serum antioxidant capacity was significantly higher in the control group than in the PTZ-receiving group (*P* < 0.001). Treatment with diazepam + extract (100 and 150 mg/kg) significantly increased serum antioxidant capacity in rats receiving PTZ (*P* <0.001). Serum antioxidant capacity was significantly lower in the flumazenil + extract (150 mg/kg)-receiving group than in the extract (150 mg/kg)-receiving group (*P* < 0.001), but there was no significant difference in serum antioxidant capacity was observed between the flumazenil + extract (150 mg/kg)-receiving group and the PTZ-receiving group (Figure 10).

### 3.11. Serum MDA level

**Figure 11.**
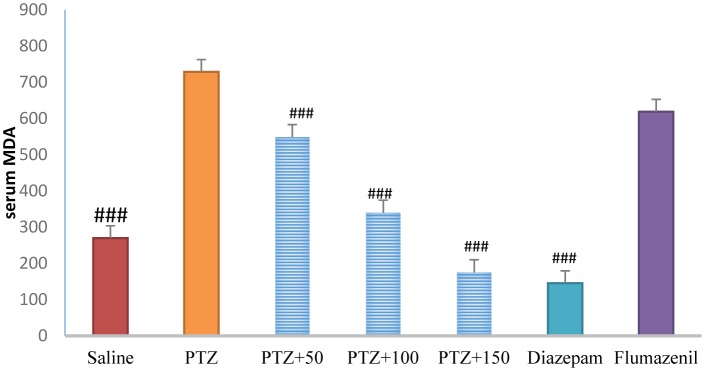
Serum malondialdehyde levels in different groups. ### Significant difference compared to pentylenetetrazol-receiving group (*P* < 0.001).

Serum MDA level was significantly lowers in the control group than in the PTZ-receiving group (*P* < 0.001). Treatment with diazepam + extract (50, 100 and 150 mg/kg) caused a significant decrease in serum MDA level in the PTZ-receiving rats (*P* < 0.001). The serum MDA level was significantly lower in the flumazenil + extract (150 mg/kg)-receiving group than in the extract (150 mg/kg)-receiving group (*P* < 0.001), but was not significantly different compared to that in the PTZ-receiving group (Figure 11).

### 3.12. Brain antioxidant capacity

**Figure 12.**
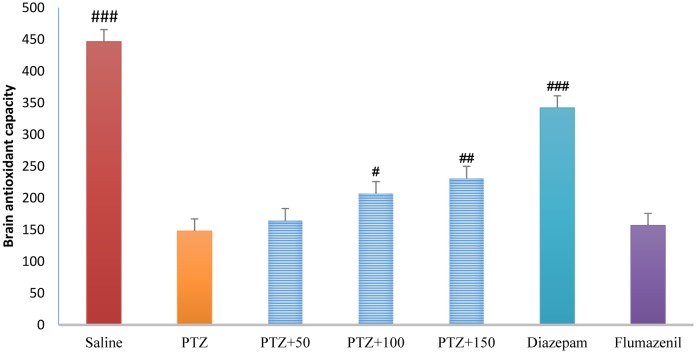
Brain antioxidant capacity in different groups. ### Significant difference compared to pentylenetetrazol-receiving group (*P* < 0.001); ## Significant difference compared to pentylenetetrazol-receiving group (*P* < 0.01); #Significant difference compared to pentylenetetrazol-receiving group (*P* < 0.05).

The antioxidant capacity was significantly higher in the control group than in the PTZ-receiving group (*P* < 0.001). Treatment with diazepam, extract (100 and 150 mg/kg) caused a significant increase in the brain antioxidant capacity in the PTZ-receiving rats (*P* < 0.01, *P* < 0.05). The brain antioxidant capacity was significantly lower in the flumazenil + extract (150 mg/kg)-receiving group than in the extract (150 mg/kg)-receiving group (*P* < 0.001), but was not significantly different compared to that in the PTZ-receiving group (Figure 12).

### 3.13. Brain MDA levels

**Figure 13.**
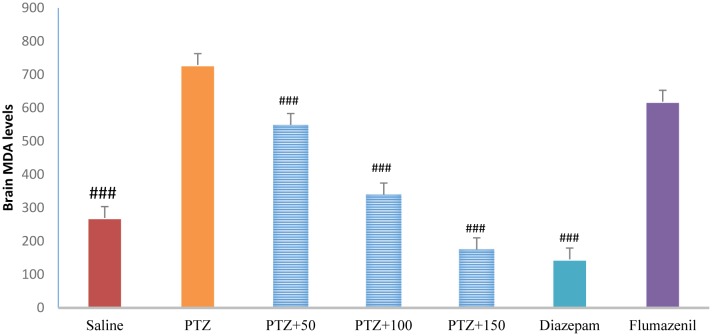
Brain malondialdehyde levels in different groups. ### Significant difference compared to pentylenetetrazol-receiving group (*P* < 0.001).

MDA was significantly lower in the control group than in the PTZ-receiving group (*P* <0.001). Treatment with diazepam, extract (50, 100 and 150 mg/kg) caused a significant decrease in brain MDA level in the PTZ-receiving group (*P* < 0.001). Brain MDA level was significantly lower in the flumazenil + extract (150 mg/kg)-receiving group than in the extract (150 mg/kg)-receiving group and the diazepam-receiving group (*P* < 0.001) (Figure 13).

## 4. Discussion

Given high prevalence of epilepsy worldwide and low efficacy of synthetic drug, it is essential to seek out effective pharmaceutical that cause comparatively fewer side effects. The present study was conducted to investigate the effect of pretreatment with hydroalcoholic extract of *A*. *officinarum* rhizome on the severity of epilepsy and memory impairment in rats with pentylenetetrazole (PTZ)-induced kindling. The results showed that *A*. *officinarum* extract (100 and 150 mg/kg) caused a significant increase in first seizure latency, and treatment with this extract at 50, 100 and 150 mg/kg significantly reduced the total frequency of the entire body seizures and frequent spinning and jumping in the PTZ-receiving rats, but did not significantly affected the total frequency of head ticks, the seizures of head and upper limbs, and tonic seizures. First seizure latency was significantly lower in the flumazenil + extract (150 mg/kg)-receiving group than in the extract (100 and 150 mg/kg)-receiving groups, but was not significantly different between the flumazenil + extract (150 mg/kg)-receiving group and PTZ-receiving group. The total frequency of the entire body seizures and frequent spinning and jumping were significantly higher in the flumazenil + extract (150 mg/kg)-receiving group than in the groups receiving extract at 50, 100 and 50 mg/kg, but was not significantly different between the flumazenil + extract (150 mg/kg)-receiving group and the PTZ-receiving group. Survival rates in rats receiving PTZ (50 mg/kg) and those treated with the extract (50, 100, and 150 mg/kg) were 90%, 100%, and 100%, respectively. In the present study, diazepam treatment increased first seizure latency and decreased the total frequency of the entire body seizures and frequent spinning and jumping significantly. Studies have reported that the GABAergic, glutaminergic and cholinergic systems are involved in epilepsy [12]. The inhibition of GABA secretion has been observed in PTZ-induced seizure. GABA agonists can reduce the severity and duration of seizures. In addition, GABA synthesis inhibitors can cause seizure. Diazepam is a GABA agonist and, as shown in previous studies, decreases the severity and symptoms of seizure in animals and humans. In the present study, treatment with flumazenil (GABA receptor antagonist) significantly inhibited the anticonvulsant effects of this extract in rats receiving *A*. *officinarum* extract (150 mg/k), which indicates that the extract activity is exerted via the GABAergic system. Acetylcholine and its nicotinic receptors are involved in the stimulation and maintenance of seizures, so that induction of epilepsy by parenteral lithium, pilocarpine, and kinate significantly increases the levels of acetylcholine in the cortex and hippocampus [3]. In addition, it has been argued that some mutations in the subtypes of nicotinic acetylcholine receptors play a role in the development of certain types of epilepsy [13]. It has been reported that the inhibition of acetylcholinesterase significantly increases the incidence rate of spontaneous seizure [14]. Köse et al. showed that acetylcholinesterase obtained from *A*. *officinarum* has anticholinergic and inhibitory properties [8]. It can be argued that the extract of the plant can reduce the severity of epileptic seizures in rats through exerting anticholinergic activity; however, this hypothesis deserves further investigation. Various compounds have been identified in *A*. *officinarum* rhizome by chromatographic techniques, including 8.1-cineol, methyl cinnamate, galangin, 3-o-methyl galangin, kaemferide, alpinin, galangol, and diarylheptanoid. Galangin and 3-O-methyl galangin are two main compounds of *A*. *officinarum* rhizome. Galangan is a flavonoid that is found in large quantities in *A*. *officinarum*rhizome. *A*. *officinarum* has anti-inflammatory and antioxidant properties [11]. Guo et al. studied the anticholinergic effects of 25 flavonoids isolated from different medicinal plants. They reported that *A*. *officinarum*, among the studied flavonoids, had the most potent effects on acetylcholinesterase [15].

The role of oxidative stress in the onset and progression of epileptic seizures has already been demonstrated [16]. In a study conducted in 50 patients with epilepsy, lipid peroxidation and hemolysis percentage of the erythrocytes were significantly higher in patients with epilepsy than in healthy subjects. The levels of glutathione reductase and vitamins C, E, and A in the erythrocytes were significantly lower in patients with epilepsy than in healthy subjects [17]. Animal studies have shown that PTZ-induced epileptic seizures lead to production of free radicals and oxidative damage to proteins, lipids, and cell DNA. The high levels of mitochondrial superoxide, the inactivation of iron and sulfur-dependent enzymes such as aconitase, and iron-induced toxicity may contribute to oxidative damage to neurons following epileptic seizures [16]. In the present study, serum and brain antioxidant capacity was significantly lower in the PTZ-receiving group than in the control group. In addition, serum and brain MDA levels were significantly higher in the PTZ-receiving group than in the control group, indicating a decrease in the serum and tissue antioxidant capacity and lipid peroxidation as well as an increase in epileptic seizures. In the present study, different concentrations of *A*. *officinarum* extract caused a significant increase in antioxidant capacity and a decrease in serum MDA levels in rats treated with PTZ. In addition, the subsequent flumazenil treatment resulted in a significant increase in lipid peroxidation and reduction in antioxidant capacity, suggesting that flumazenil inhibits damage to the neurons by preventing anticonvulsant effects of the plant. In addition, studies have reported that *A*. *officinarum* and 3-o-methyl galangin are two major compounds of *A*. *officinarum* rhizome with antioxidant properties [11]. Experimental studies have shown that the induction of epilepsy in rodents causes a significant increase in inflammation mediators in the epilepsy-affected regions [18],[19]. High levels of inflammatory cytokines, such as tumor necrosis factor alpha and interleukin-6, in the astrocytes can reduce seizure threshold and the frequency of spontaneous seizures. Experimental studies on animal models have revealed that anti-inflammatory drugs can reduce the severity of certain types of epilepsy [19]. It was reported that the levels of inflammatory cytokines including tumor necrosis factor alpha, interleukin-6, and interleukin-1 beta as well as interleukin-1 receptor antagonist significantly increased in patients with epilepsy [20]. The compounds with anti-inflammatory effects can help decrease the severity of epileptic seizures. The anti-inflammatory effects of *A*. *officinarum* have already been demonstrated. For example, Yadav et al. reported that *A*. *officinarum* extract treatment significantly reduced nitric oxide production due to stimulation by lipopolysaccharide in macrophage cell line in rats [21]. Treatment of the mononuclear cells of human peripheral blood with *A*. *officinarum* extract significantly reduced the production of the inflammatory cytokines interleukin-1 beta and tumor necrosis factor alpha due to stimulation by lipopolysaccharide [22]. The study of Lee et al. showed that the ethanolic extract of *A. officinarum* rhizome significantly reduced edema volume in carrageenan-induced arthritis model [21]. Studies have shown that galangin found in *A*. *officinarum* rhizome has anti-inflammatory effects [11]. It can be argued that *A*. *officinarum* extract prevents damage to the neurons and associated disorders by decreasing the levels of inflammation mediators. In the present study, in the shuttle box test, the secondary latency was significantly higher in the control group than in the PTZ-receiving group, suggesting a decline in passive avoidance memory in seizure in rats. Treatment with diazepam and *A*. *officinarum* (100 and 150 mg/kg) in the PTZ-receiving rats caused a significant increase in the secondary latency. Cognitive disorders frequently occur in patients with epilepsy. In addition, epilepsy can disrupt cognitive processes; common antiepileptic drugs also disrupt cognitive processes, which lead to a significant decline in quality of life. Epileptic seizures are associated with damage to neurons in the limbic system including CA3, CAL, hippocampal torsion, amygdala, and entorhinal cortex. Damage to the neurons in the hippocampus causes memory and learning impairment. The role of the hippocampus as a system involved in memory processes has been well explained, and damage to this region of the brain can cause severe forgetfulness. Agarwal et al. reported that PTZ-induced epilepsy in rats caused impairment of passive avoidance memory and significantly reduced latency to enter the dark chamber in the shuttle box test, which is consistent with our results. In addition, studies have reported that PTZ-induced epileptic seizures cause spatial memory impairment, learning disorders, and passive avoidance memory in laboratory animals [23]. In the present study, *A*. *officinarum* extract significantly improved passive avoidance memory in rats receiving PTZ. It can be argued that galangan extract prevents memory loss by preventing damage to tissues involved in memory and learning. Yongfang et al. reported that *A*. *officinarum*, which is the main flavonoid compound of *A*. *officinarum* rhizome, significantly prevented memory loss and passive avoidance memory due to d-galactose. They reported that *A*.*officinarum* exerted protective effects on the central nervous system by reducing oxidative stress parameters and increasing the activity of the NA^+^-ATPase and K^+^-ATPase channels [24]. In the present study, immobility time in the TST was significantly lower in the control group than in the PTZ-receiving group, suggesting the induction of depression due to epileptic seizures. In addition, treatment with diazepam and *A*. *officinarum* extract (50, 100 and 150 mg/kg) significantly reduced the immobility time in the PTZ-receiving rats. It has been reported that the prevalence of depression is significantly higher in patients with epilepsy than in the general population [24]. Depression is a serious healthcare and social problem in patients suffering from epilepsy that affects their quality of life. Depression affects almost half of the patients under treatment for epilepsy. It has been reported that loss of occupation and social activity exacerbates depression in patients with epilepsy [25]. We choose diazepam as a positive control group (anstablished drug used to treat epilepsy with partially determined underlying mechanism of action mostly via GABAergic system. There are several pattern and mechanism of the seizure and agent may affected one or more pathway based on this affect the potency and efficacy of favorite agent are difficult. The present work is the first work to show that PTZ treatment in rats caused depression-related behaviors, and treatment with *A*. *officinarum* extract caused a significant improvement of these behaviors. According to our findings, *A. officinarum* extract can be used as an adjuvant agent along with commercial drugs to treat epilepsy.

## 5. Conclusion

According to the results of the current study, treatment with *A*. *officinarum* extract in the PTZ-receiving rats significantly increased first seizure latency and reduced the frequency of the entire body seizures and frequent spinning and jumping. Flumazenil treatment in *A*. *officinarum* extract (150 mg/kg)-receiving rats significantly inhibited anticonvulsant effects of the extract. Treatment with *A*. *officinarum* extract in PTZ-receiving rats significantly increased passive avoidance memory in the shuttle box test and reduced immobility time in the TST. *A*. *officinarum* extract can be used to reduce the severity of epileptic seizures and improve learning disorders and associated mood disorders.
